# Circulating Growth Differentiation Factor 15 Levels Are Associated With Risk of Both Intracerebral and Subarachnoid Hemorrhage

**DOI:** 10.3389/fneur.2021.664010

**Published:** 2021-06-09

**Authors:** Lu Song, Martin Söderholm, Edith H. Svensson, Yan Borné, Gunnar Engström

**Affiliations:** ^1^Department of Neurology, Xinhua Hospital, Shanghai Jiaotong University School of Medicine, Shanghai, China; ^2^Department of Clinical Sciences, Lund University, Malmö, Sweden; ^3^Department of Neurology, Skåne University Hospital, Malmö, Sweden

**Keywords:** growth differentiation factor 15, intracerebral hemorrhage, subarachnoid hemorrhage, risk factor, population-based study

## Abstract

**Background:** Growth differentiation factor 15 (GDF-15) has been associated with the risk of developing major bleedings, including but not restricted to intracranial hemorrhages, in patients on oral anticoagulants or dual antiplatelet therapy. We hypothesized that there may be an association of GDF-15 with incidence of hemorrhagic strokes in the general population, which has not been investigated before.

**Methods:** Two different case-control studies, one for intracerebral hemorrhage (ICH) and one for subarachnoid hemorrhage (SAH), nested within the population-based Malmö Diet and Cancer cohort, were defined using the incidence density sampling method. GDF-15 was analyzed in frozen blood samples taken at the baseline examination in 1991–1996. The associations between GDF-15 and incident ICH (220 cases, 244 controls) and incident SAH (79 cases, 261 controls), respectively, were explored using conditional logistic regression adjusting for risk factors.

**Results:** GDF-15 levels at baseline were higher in both incident ICH and SAH cases, compared with their respective control subjects. After adjustment for risk factors, significant relationships with high GDF-15 concentrations were observed both for incident ICH (odds ratio (OR) per 1 log2 unit: 2.27, 95% confidence interval (CI): 1.52–3.41; *P* = 7.1 × 10^−5^) and incident SAH (OR: 2.16, 95% CI: 1.29–3.59; *P* = 0.0032).

**Conclusions:** High circulating GDF-15 levels were associated with incident ICH and incident SAH, independently of the main risk factors.

## Introduction

Spontaneous intracerebral hemorrhage (ICH) and subarachnoid hemorrhage (SAH) together account for about 15–25% of all strokes (1). Although less common than ischemic stroke, ICH and SAH are associated with high mortality rates and severe sustained disability (2, 3). As there are few specific interventions to improve outcome after ICH and SAH (4, 5), better prediction and prevention of ICH and SAH is of great importance.

Growth differentiation factor 15 (GDF-15), a stress-responsive member of the transforming growth factor cytokine superfamily, is a marker of cellular aging, cellular growth, oxidative stress, and inflammation (6). Generally, the production of GDF-15 is low in most human tissues, except for the placenta (7). However, in pathological situations, such as oxidative stress, hypoxia, inflammation, and tissue injury, the expression of GDF-15 can be substantially increased in many cell types (8).

GDF-15 has been found to be an independent risk factor for a composite endpoint of major bleeding events in patients with atrial fibrillation on oral anticoagulants and in patients with acute coronary syndrome on dual antiplatelet therapy (9–11). The association of GDF-15 with bleeding events may partly be attributed to its inhibitory effect on platelet integrin activation and thrombus formation (12). Also, GDF-15 is believed to mirror vascular endothelium damage caused by oxidative stress and inflammation (13). Previous population-based studies have found that circulating GDF-15 levels are associated with cardiovascular risk factors such as age, current smoking, diabetes, and hypertension (14). However, the association between GDF-15 and incidence of hemorrhagic strokes has not, to our knowledge, been specifically investigated. In the present study, we evaluated the association between circulating GDF-15 levels and incident ICH and SAH, respectively, in community-dwelling middle-aged individuals from a population-based study.

## Method

### Study Population

The Malmö Diet and Cancer (MDC) study is a large prospective population-based cohort performed in Malmö, Sweden. Details of the MDC study have been published (15). Briefly, 28,449 individuals (11,246 men and 17,203 women), born between 1923 and 1950, participated in the baseline examinations between 1991 and 1996. Informed consent was obtained from all participants, and the study was approved by the ethics committee at Lund University (LU51/90,166/2007, 633/2009, 566/2013, 2016/452).

### Baseline Examinations

The examinations were carried out according to standard procedures. Blood pressure was measured in the supine position after 10 min rest. Body mass index and waist circumference were measured. Information about smoking habits, alcohol intake, medical history, medications, and lifestyle factors was obtained from a self-administered questionnaire. Individuals who smoked regularly or occasionally were classified as current smokers, and former or never smoker were classified as non-smokers. High alcohol intake was defined as >40 g/day for men and >30 g/day for women.

### Outcome

All participants were followed up until an incident ICH or SAH event, death, emigration from Sweden, or December 31, 2010. The local stroke register of Malmö and the national Swedish Hospital Discharge and Causes of Death Registries were used to identify the cases of first-ever ICH and SAH (16–18).

The ICH diagnosis was based on intra-parenchymal bleeding in the brain revealed by computed tomography, magnetic resonance imaging, or autopsy. If secondary causes of ICH (here defined as, trauma, tumor, arteriovenous malformation, or hemorrhagic infarction) were identified, the case was excluded. The ICHs were categorized based on location as lobar (mainly cortical or subcortical regions affected) or non-lobar (basal ganglia, internal capsule, periventricular white matter, cerebellum, or brain stem) by a neuroradiologist (16). The formula A × B × C/2 (19) was used to assess the ICH volume. From the Swedish quality register for stroke, Riksstroke, information of functional outcome 3 months after ICH was obtained. This information was converted into the modified Rankin Scale (mRS) (20). According to mRS, a score between 0 and 6 is assigned, with 0 indicating no symptoms, 3 is moderate disability, and 6 is the score for fatal cases.

The SAH diagnosis was based on bleeding into the subarachnoid space revealed by computed tomography, lumbar puncture, magnetic resonance imaging, or autopsy. Medical records, including patient history and imaging, were validated to exclude traumatic SAH cases (18).

### Nested Case-Control Studies

**Figure 1:**
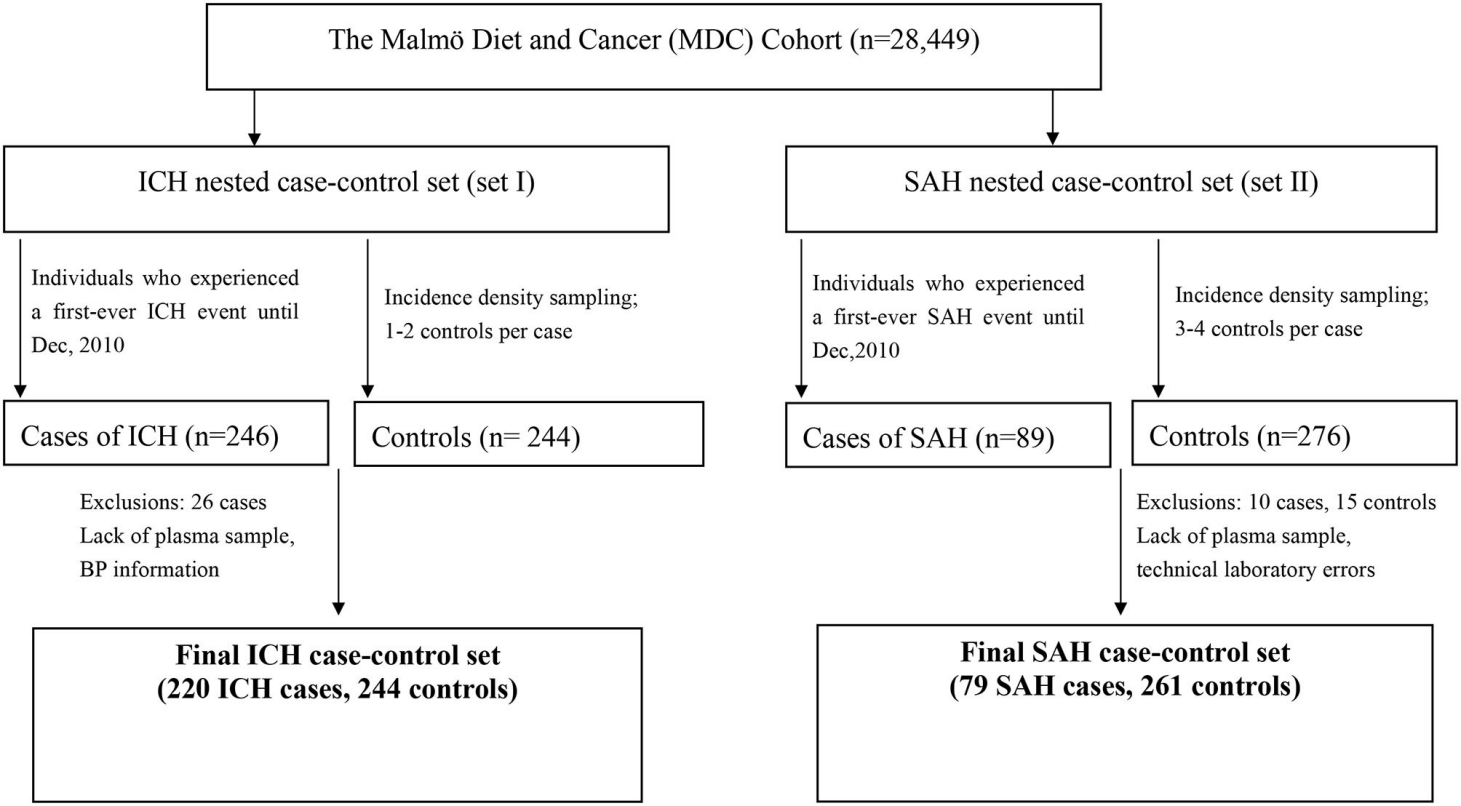
Participants included in the nested case-control studies of GDF-15 levels and ICH and SAH.

Two different nested case-control sets from the MDC cohort were used in the present study. Controls were selected randomly from the MDC using incidence density sampling, matching on age and sex, excluding subjects with a history of ICH in the ICH case-control set, and with a history of SAH in the SAH case-control set (17, 18) (Figure 1).

ICH cases were individuals who experienced a first-ever ICH event during follow-up (incident ICH). The date when ICH was diagnosed was considered the index date. For each ICH case, one to two controls still at risk for a first ICH on the index date were sampled, matched for sex and age. There were 246 first-ever incident ICH cases in the MDC Study up to December 2010. Twenty-five cases were excluded because of missing plasma samples or laboratory errors; one case was excluded because blood pressure information was missing. Eventually, 220 ICH cases [mean age (+SD) 62 years (+7), 48% men] and 244 control subjects [mean age 62 years (+7), 49% men] were included (Table 1). The mean time from the baseline examination to the ICH event was 9.2 years (SD: 4.7, range: 0.05–19.3).

**Table 1:** Baseline characteristics including levels of GDF-15 in ICH cases and controls.

	**All**	**Controls**	**Cases**	***P* value**
*n*	464	244	220	
Age (year, mean ± SD)	62 ± 7	62 ± 7	62 ± 7	0.88
Men [*n* (%)]	224 (48)	119 (49)	105 (48)	0.82
Systolic blood pressure (mmHg, mean ± SD)	149 ± 21	146 ± 21	153 ± 20	0.0012
Diastolic blood pressure (mmHg, mean ± SD)	88 ± 11	87 ± 11	90 ± 11	0.0028
Blood pressure medication [*n* (%)]	111 (24)	55 (23)	56 (25)	0.46
Body mass index (kg/m^2^, mean ± SD)	25.9 ± 17.9	25.8 ± 3.9	26.0 ± 4.2	0.56
Diabetes mellitus [*n* (%)]	28 (6.0)	14 (5.7)	14 (6.4)	0.78
Smoking [*n* (%)]	119 (26)	49 (20)	70 (32)	0.004
High alcohol consumption^*^ [*n* (%)]	20 (4.3)	6 (2.5)	14 (6.4)	0.039
Use of warfarin [*n* (%)]	10 (2.2)	2 (0.8)	8 (3.6)	0.037
GDF-15^+^, mean ± SD (log2 transformed)	8.07 (0.78)	7.94 (0.77)	8.20 (0.77)	0.0003
GDF-15^+^ (median interquartile range)	263 (176–370)	236 (165–399)	298 (205–417)	0.0001^**^

*GDF, growth differentiation factor 15*.

* *>40 g/day for men and >30 g/day for women*.

+ *Normalized protein expression (NPX) values*.

*P-values for cases vs. controls were calculated using ANOVA or Pearson χ2*.

** *Mann-Whitney U test used due to skewed distribution of GDF15 values*.

Similarly, another independent nested case-control study including SAH cases and controls, matched to the cases by sex and age and follow-up time (three to four controls per case), was constructed (18). For SAH, 89 incident cases in the MDC Study up to December 2010 were identified for the present study and 276 controls were selected. Ten cases and 15 control subjects were excluded for technical laboratory errors or insufficient plasma samples for analysis. Seventy-nine SAH cases [mean (+SD) age 58 years (+8), 28% men] and 261 control subjects [mean age 58 years (+8), 26% men] remained for analysis. From the baseline examination to the SAH event, the mean time interval was 8.5 years (SD: 4.6, range: 0.3–17.5).

### Laboratory Analysis

Blood samples were obtained at the baseline examination and were stored at −80°C until analysis. The concentration of GDF-15 was measured in plasma for the whole ICH case-control set in 2015 and in serum for the whole SAH case-control set in 2014. Previous study has indicated the long-term storage of baseline sample is sufficiently stable for the analysis (21). The laboratory analysis of GDF-15 was performed at the Science for Life Laboratory (Uppsala, Sweden) using the Proseek Multiplex CVD I^96×96^ reagent kit (Olink Proteomics, Uppsala, Sweden) on 96-sample plates (22). Proximity extension assay technology was used for the high-specificity Proseek assay (23, 24). The intra- and interassay coefficients of variation for GDF-15 were 9 and 11% (25) GDF-15 was log2 transformed to achieve normal distributions. The GDF-15 concentrations measured with this method have been shown to correlate well with concentrations measured by a electrochemiluminescence immunoassay (Roche Diagnostics, Mannheim, Germany) (*r* = 0.89) (21).

### Statistical Analysis

Analyses were performed for GDF-15 in quartiles and as a continuous variable, per 1 unit of the log2 value, which corresponds to a doubled concentration of GDF-15. The associations between GDF-15 and incident ICH and incident SAH, respectively, were explored using conditional logistic regression, conditioned for the 96-well plate used for GDF-15 measurement, and adjusting for age and sex. Additional models, adjusting for systolic blood pressure, body mass index, current smoking, diabetes mellitus, high alcohol consumption, blood pressure medication, and use of warfarin were also performed. Separate analyses were performed to evaluate the risk for specific ICH categories; lobar and non-lobar ICH, fatal ICH (within 28 days), ICH with large volume (≥40 ml), and ICH with poor functional outcome (mRS, 3–6 at 3 months). We also evaluated the association between GDF-15 and ICH occurring in the first 10 years from baseline and after 10 years of follow-up, respectively. A *P* < 0.05 was considered statistically significant.

### Data Availability

The data underlying this study are owned by Lund University. Data are available upon reasonable request after application to the MDC Study steering committee.

## Results

### Baseline Characteristics

Baseline characteristics and GDF-15 levels are shown in Table 1 for the ICH case-control dataset and in Table 2 for the SAH case-control set. Both incident ICH and SAH cases had higher GDF-15 levels compared with their respective control groups. In ICH cases, systolic blood pressure was higher, and current smoking, high alcohol intake, and warfarin use were more common, compared with the control subjects (all *P* < 0.05). SAH cases had a higher prevalence of smoking and higher blood pressure compared with control subjects (both *P* < 0.05).

**Table 2:** Baseline characteristics including levels of GDF-15 in SAH cases and controls.

	**All**	**Controls**	**Cases**	***P* value**
*n*	340	261	79	
Age (year, mean ± SD)	58 ± 8	58 ± 8	58 ± 8	0.72
Men [*n* (%)]	90 (26)	68 (26)	22 (28)	0.75
Systolic blood pressure (mmHg, mean ± SD)	139 ± 19	138 ± 19	143 ± 18	0.020
Diastolic blood pressure (mmHg, mean ± SD)	85 ± 9.5	84 ± 9.5	87 ± 9.0	0.0090
Blood pressure medication [*n* (%)]	50 (15)	34 (13)	16 (20)	0.11
Body mass index (kg/m^2^, mean ± SD)	25.0 ± 3.8	25.0 ± 3.6	24.7 ± 4.3	0.54
Diabetes mellitus [*n* (%)]	8 (2.4)	5 (1.9)	3 (3.8)	0.33
Smoking [*n* (%)]	111 (33)	71 (27.0)	40 (51)	<0.0001
High alcohol consumption^*^ [*n* (%)]	12 (3.5)	11 (4.1)	1 (1.3)	0.21
Use of warfarin [*n* (%)]	3 (1.0)	1 (0.4)	2 (2.5)	0.074
GDF-15^+^ [mean ± SD (log2 transformed)]	9.61 ± 0.59	9.53 ± 0.55	9.85 ± 0.65	0.00003
GDF-15^+^ (median interquartile range)	739 (416)	724 (376)	942 (518)	0.0001^**^

*GDF, growth differentiation factor 15*.

* *>40 g/day for men and >30 g/day for women*.

+ *Normalized protein expression (NPX) values*.

*P-values for cases vs. controls were calculated using ANOVA or Pearson χ2*.

** *Mann-Whitney U test used due to skewed distribution of GDF15 values*.

### Intracerebral Hemorrhage

Sixty-eight of the 220 ICH cases (31%) were fatal within 28 days. Among 183 patients with known ICH location, 82 cases were lobar ICH and 101 were non-lobar ICH. Of 166 patients with known ICH volume (median volume, 14 ml; range: 0.14–254 ml; interquartile range: 4–45 ml), 41 cases had a volume ≥40 ml. One hundred sixty-six ICH cases had information about mRS at 3 months; 135 of them had poor outcome (mRS 3–6).

**Figure 2:**
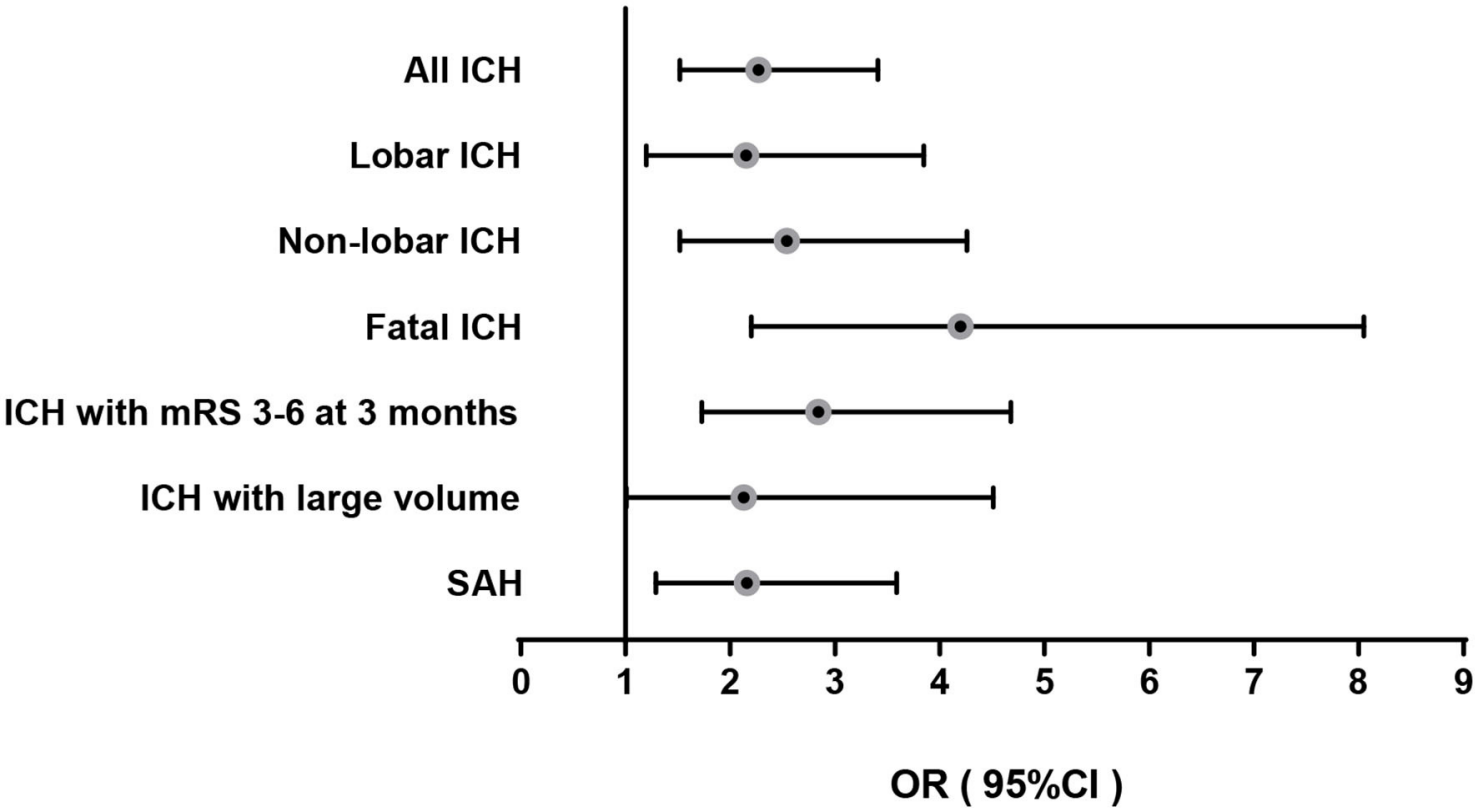
Multivariable-adjusted odds ratio for ICH and SAH per 1 log unit increase of GDF-15.

Plasma GDF-15 at baseline was associated with incident ICH adjusting for age and sex, and after adjustment in the multivariable model (odds ratio (OR) per 1 log unit: 2.27, 95% confidence interval (CI): 1.52–3.41; *P* = 7.1 × 10^−5^; Table 3). There were significant associations between GDF-15 and both lobar ICH (multivariable-adjusted OR: 2.15; 95% CI: 1.20–3.85) and non-lobar ICH (OR: 2.54; 95% CI: 1.52–4.26, Table 3). In addition, GDF-15 was related to incident fatal ICH (multivariable-adjusted OR: 4.20; 95% CI: 2.20–8.05; *P* = 1.5 × 10^−5^), ICH with poor functional outcome at 3 months (OR: 2.84; 95% CI: 1.73–4.68; *P* = 4.0 × 10^−5^), and ICH with large volume (OR: 2.13; 95% CI: 1.01–4.51; *P* = 0.048) (Figure 2).

**Table 3:** Association between GDF-15 and risk of ICH and ICH subtypes.

	**Quartiles of GDF-15**						
	**Q1**	**Q2**	**Q3**	**Q4**	***P* for trend**	**GDF-15 per 1 unit**	***P* values**
*N* (all)	116	116	116	116			
*n* (ICH cases)	40	54	56	70			
GDF-15^+^ [mean (range)]	7.12 (6.41–7.45)	7.76 (7.46–8.04)	8.29 (8.04–8.53)	9.09 (8.53–10.6)			
**All ICH**							
Age- and sex-adjusted OR (95% CI)	1	1.99 (1.14–3.49)	2.80 (1.47–5.33)	5.99 (2.85–12.6)	3.4 × 10^−6^	2.38 (1.63–3.46)	6.3 × 10^−6^
Multivariable-adjusted OR^*^ (95% CI)	1	2.05 (1.15–3.66)	2.54 (1.29–5.02)	5.74 (2.56–12.8)	4.2 × 10^−5^	2.27 (1.52–3.41)	7.1 × 10^−5^
**Lobar ICH**							
Age- and sex-adjusted OR (95% CI)	1	3.44 (1.49–7.98)	3.32 (1.22–9.00)	7.46 (2.47–22.5)	0.0009	2.22 (1.30–3.79)	0.0035
Multivariable-adjusted OR^*^ (95% CI)	1	3.59 (1.53–8.45)	3.17 (1.31–8.87)	7.48 (2.29–24.4)	0.0020	2.15 (1.20–3.85)	0.010
**Non-lobar ICH**							
Age- and sex-adjusted OR (95% CI)	1	1.28 (0.61–2.29)	2.21 (0.96–5.07)	5.37 (2.15–13.4)	0.0003	2.38 (1.50–3.76)	0.0002
Multivariable-adjusted OR^*^ (95% CI)	1	1.38 (0.63–3.01)	2.08 (0.86–5.08)	5.35 (1.95–14.7)	0.0012	2.54 (1.52–4.26)	0.0004

*CI, confidence interval; ICH, intracerebral hemorrhage; OR, odds ratio; GDF-15, growth differentiation factor 15*.

+ *Normalized protein expression (NPX) values on log2 scale*.

* *Adjusted for age, sex, systolic blood pressure, blood pressure medication, body mass index, current smoking, diabetes mellitus, high alcohol consumption, and use of warfarin*.

GDF-15 levels were associated with ICH incidence both in the first 10 years of follow-up (*n* cases = 115) and after 10 years of follow-up (*n* cases = 105); the ORs per 1 log unit increase adjusted in the multivariable model were 2.66 (95% CI: 1.62–4.35, *P* = 0.00010) and 1.99 (95% CI: 1.17–3.37, *P* = 0.011), respectively.

### Subarachnoid Hemorrhage

High GDF-15 levels at baseline were significantly associated with incident SAH, and the results remained after multivariable adjustment (Figure 2 and Table 4). The multivariable-adjusted OR for SAH per one log unit increase of GDF-15 was 2.16 (95% CI: 1.29–3.59, *P* = 0.0032).

**Table 4:** Association between GDF-15 and risk of SAH.

	**Quartiles of GDF15**						
	**Q1**	**Q2**	**Q3**	**Q4**	***P* for trend**	**GDF-15 per 1 unit**	***P* values**
*N* (all)	88	83	85	84			
*n* (SAH cases)	12	15	20	32			
GDF15^+^ [mean (range)]	8.93 (8.52–9.18)	9.38 (9.19–9.53)	9.75 (9.54–9.96)	10.39 (9.97–12.13)			
Age- and sex-adjusted OR (95% CI)	1	1.48 (0.64–3.43)	2.21 (0.97–5.01)	4.90 (2.15–11.1)	7.5 × 10^−5^	2.76 (1.70–4.50)	4.3 × 10^−5^
Multivariable-adjusted OR^*^ (95% CI)	1	1.21 (0.51–2.87)	1.77 (0.76–4.13)	3.23 (1.34–7.76)	0.0054	2.16 (1.29–3.59)	0.0032

*CI, confidence interval; SAH, subarachnoid hemorrhage; OR, odds ratio; GDF-15, growth differentiation factor 15*.

* *Adjusted for systolic blood pressure, blood pressure medication, current smoking, diabetes mellitus, body mass index, high alcohol intake, and use of warfarin*.

+ *Normalized protein expression (NPX) values on Log2 scale*.

## Discussion

In the present study, the associations of GDF-15 with incident ICH and SAH were explored using two different nested case-control sets from a population-based cohort study. Higher circulating concentrations of GDF-15 were associated with both incident ICH and incident SAH, even after adjusting for the major established risk factors. The risk was significantly increased both for ICH with lobar and non-lobar location and also for ICH with fatal or poor functional outcome. The results are in accordance with previous data from studies linking GDF-15 to the overall risk of major bleeding events in patients with anticoagulants or dual antiplatelet therapy. However, this is the first study to specifically show associations of GDF-15 levels with spontaneous incident hemorrhagic strokes in the general population.

GDF-15 is a stress-induced cytokine and can be released from macrophages, vascular smooth muscle cells, cardiomyocytes, adipocytes, and endothelial cells in response to various pro-inflammatory cytokines (6). Although the exact GDF-15 receptor and the involved signaling pathway is not fully clarified, GDF-15 is recognized as a multifunctional protein involved in prenatal development, inflammation, modulation of cellular stress responses, and tissue repair after acute injuries (8, 26).

Some studies have indicated a potential use of GDF-15 as a clinical biomarker for predicting overall cardiovascular disease risk (14). GDF-15 was also shown to be related to the risk of developing major bleedings in patients with atrial fibrillation on oral anticoagulants and in patients with acute coronary syndrome on dual antiplatelet therapy, respectively, but with inconsistent results for the relationship of GDF-15 with all-cause stroke (9–11, 27). Major bleedings were defined as symptomatic bleedings across different organs, including intracranial hemorrhage, and also significant hemoglobin reduction and other bleedings requiring blood transfusion. The relationships between GDF-15 and risk of intracranial hemorrhages, ICH or SAH were not separately evaluated in those studies.

Another study indicated that GDF-15 is an independent predictor of all-cause stroke and ischemic stroke (*n* = 12), but not ICH, in 254 patients with hypertension (28). However, only 10 ICH cases were included in this study, and the negative results could be due to a beta error. For community-dwelling individuals, GDF-15 was independently related to the composite endpoint of major cardiovascular events, including all-cause stroke (29, 30) and in another study, GDF-15 was associated with atherothrombotic stroke (31). The specific association between GDF-15 and incident hemorrhagic stroke has not previously been studied in the general population, to our knowledge.

Although ICH and SAH are different disease entities, they share some risk factors. Common risk factors for ICH and SAH, such as age, hypertension, and smoking, are also related to circulating levels of GDF-15 in elderly individuals (14). To what extent increased GDF-15 is caused by these cardiovascular risk factors is not known, but studies have shown these risk factors account for only a proportion of variation of GDF-15 among individuals, suggesting GDF-15 carries additional information (14). Furthermore, the results in the present study were still strong after adjusting for the major known risk factors for ICH and SAH.

There are several biological effects of GDF-15 that potentially could increase the risk of ICH and SAH over long time. Fluid dynamics and in?ammatory remodeling of the blood vessels play important roles in the formation and rupture of aneurysms (32), which cause 85% of all SAHs. ICH is usually caused by rupture of small penetrating arteries secondary to hypertensive small vessel disease or other vasculopathies, which also involves damage of the vascular endothelium (2). GDF-15 is produced by endothelial cells in response to vascular stress and could then further participate in modulation of the vascular response to injury, such as regulating apoptotic cell death, IL-6-dependent inflammatory reactions, and function of vascular progenitor cells (33, 34). These mechanisms of GDF-15 could hypothetically increase the risk of both ICH and SAH, for example, by contributing to small vessel disease or inflammatory remodeling in aneurysms. Furthermore, the inhibitory effect of GDF-15 on platelet integrin activation and thereby thrombus formation (12) may be another mechanism by which GDF-15 could contribute to an increased risk of bleeding and hence possibly to an increased risk of ICH and SAH in individuals with small vessel disease or aneurysms.

The mechanisms of GDF-15 may also partly be mediated by conditions that are developed during follow-up. For example, GDF-15 is also associated with increased risk of incident diabetes (21), which is also a risk factor for non-lobar ICH (35), and this could hypothetically explain part of the association with non-lobar ICH. However, a recent genome-wide association study (GWAS) showed that GDF-15 was not associated with type 2 diabetes or glycemic traits (36). Moreover, diabetes is not an established risk factor for lobar ICH and SAH, which makes it likely that other effects of GDF-15 are important.

The strengths of this study include the nested case-control design with blood samples collected before the occurrence of the hemorrhagic stroke events in a large prospective population-based cohort of middle-aged individuals. Detailed individual risk factor data was available and allowed for adjustment of potential confounders. The majority of the ICH and SAH cases were validated by medical records and images, and both local and nationwide register were used to retrieve cases and therefore the loss-to-follow-up was virtually none.

There are also some limitations. GDF-15 was measured using a proximity extension assay method, which uses two DNA-labeled antibodies for two adjacent epitopes on the GDF-15 molecule. The method is very specific, but results are given as arbitrary relative units and not as absolute values in milligrams per liter. This prevents direct comparison with levels from other studies. However, the correlation between GDF-15 concentrations measured by this method and by a electrochemiluminescence immunoassay is very high (*r* = 0.89), supporting the validity of the present method (21).

Another potential limitation is that GDF-15 was measured only once in baseline blood samples that had been frozen and stored many years before analysis. This may raise the question whether GDF-15 levels remain stable in blood samples over time. However, it has previously been shown that circulating GDF-15 levels in blood samples taken 5 years apart but analyzed at the same time several years later, correlated strongly (37). Also, in another prospective cohort study, GDF-15 levels in blood samples collected and stored 14–18 years before GDF-15 measurements, had a strong predictive ability of worse outcome after cardiovascular and non-cardiovascular diseases, supporting that GDF-15 levels measured in samples stored for a long time are stable over time and could still be related to disease outcome after many years (38).

The time between baseline when blood samples were drawn and the ICH and SAH events varied between 0.05 and 19.3 years, and long follow-up time is necessary to achieve a sufficient number of cases of hemorrhagic stroke for this kind of studies. We found that the effect of GDF-15 was similar or only somewhat lower after 10 years compared with the first 10 years of follow-up, supporting long-term effects of GDF-15. Furthermore, we cannot exclude residual confounding from measurements of cardiovascular risk factors. Finally, a predominantly white (Northern European) population was included in this study, so it would be important to replicate our results in populations of other ethnicities.

In conclusion, high GDF-15 is associated with incident ICH and incident SAH after adjusting for the major known risk factors. Further studies evaluating the potential utility of adding GDF-15 for clinical prediction of hemorrhagic stroke risk in certain situations would be of great interest.

## Data Availability Statement

The original contributions presented in the study are included in the article/supplementary material, further inquiries can be directed to the corresponding authors.

## Ethics Statement

The studies involving human participants were reviewed and approved by ethics committee at Lund University (LU51/90, 166/2007, 633/2009, 566/2013, and 2016/452). The patients/participants provided their written informed consent to participate in this study.

## Author Contributions

LS and MS were responsible for study design, data analysis, and writing the manuscript for intellectual content. YB and GE were responsible for study design and critical review. ES gave critical review. All authors contributed to the article and approved the submitted version.

## Conflict of Interest

The authors declare that the research was conducted in the absence of any commercial or financial relationships that could be construed as a potential conflict of interest.
